# The NAD salvage pathway enzyme NMNAT-C sustains dark-phase NAD^+^ homeostasis in cyanobacteria

**DOI:** 10.1093/plphys/kiag143

**Published:** 2026-03-16

**Authors:** Feng Zhang, Hailei Zhang, Pengxi Wang, Yinyao Qi, Huankai Li, Lin Zhu, Gefei Huang, Yiji Xia, Zongwei Cai

**Affiliations:** Department of Chemistry, Hong Kong Baptist University, Hong Kong SAR, China; Department of Biology, Hong Kong Baptist University, Hong Kong SAR, China; College of Biological and Environmental Sciences, Zhejiang Wanli University, Ningbo 315100, China; Department of Biology, Hong Kong Baptist University, Hong Kong SAR, China; Department of Biology, Hong Kong Baptist University, Hong Kong SAR, China; Department of Chemistry, Hong Kong Baptist University, Hong Kong SAR, China; Department of Chemistry, Hong Kong Baptist University, Hong Kong SAR, China; Department of Chemistry, Hong Kong Baptist University, Hong Kong SAR, China; Department of Biology, Hong Kong Baptist University, Hong Kong SAR, China; College of Biological and Environmental Sciences, Zhejiang Wanli University, Ningbo 315100, China; State Key Laboratory of Agrobiotechnology, The Chinese University of Hong Kong, Hong Kong SAR, China; AoE Centre for Plant Vacuole Biology and Biotechnology, The Chinese University of Hong Kong, Hong Kong SAR, China; Department of Chemistry, Hong Kong Baptist University, Hong Kong SAR, China; Eastern Institute for Advanced Study, Eastern Institute of Technology, Ningbo 315200, China

## Abstract

Nicotinamide adenine dinucleotide (NAD^+^) is a crucial cofactor in cyanobacteria, which serve as model organisms for studying photosynthesis. Maintaining NAD^+^ homeostasis in cyanobacteria is critically important, and it is currently believed that multiple pathways contribute to NAD^+^ biosynthesis in these organisms. However, the specific contribution of each pathway to NAD^+^ supplementation under both light and dark conditions, which determines NAD^+^ homeostasis, has not yet been studied. In this study, we identified NMNAT-C, a cyanobacterial nicotinamide nucleotide adenylyltransferase (NMNAT), as a key player in NAD^+^ homeostasis, particularly during dark phases. NMNAT-C showed opposite-phase oscillations in expression, aligned with NAD^+^ fluctuations during light–dark cycles. Genetic and biochemical tests revealed that deleting NMNAT-C in one cyanobacterium (*Synechococcus elongatus* PCC 7942) accelerated NAD^+^ depletion during dark periods, increased sensitivity to dark stress, and impacted growth rate. Conversely, induced overexpression of NMNAT-C temporarily raised NAD^+^ levels but also caused adverse effects over time. Metabolomic analysis indicated that NMNAT-C plays a role in mediating the metabolic crosstalk between the NAD^+^ salvage pathway and the de novo pathway. Our results identify NMNAT-C as a key regulator of NAD^+^ dynamics that aligns with daily cycles and suggest that this enzyme plays a crucial role in maintaining NAD^+^ homeostasis through the NAD^+^ salvage pathway.

## Teaser

A recycling enzyme helps cyanobacteria maintain levels of a vital cellular cofactor during darkness, supporting metabolic stability across daily light–dark cycles.

## Introduction

Cyanobacteria are ancient photosynthetic bacteria that play a vital role in the global carbon cycle and oxygen production. As the only prokaryotes capable of capturing light energy similarly to plants, they are model organisms for basic research, such as for studying photosynthesis. In addition, they are widely used in synthetic biology to produce high-value compounds (Gao et al. 2012; Kanno et al. 2017; Schuergers et al. 2017). To improve the economic viability of bioproduction, extensive research has focused on engineering coenzyme metabolism to boost reducing power, particularly NADH and NADPH (Meng et al. 2021; Gao et al. 2023). Elucidating the molecular basis of NAD(P)(H) metabolism in cyanobacteria is essential for developing strategies to improve applied research. It will also provide valuable insights into NAD^+^ metabolism in higher plant species.

NAD^+^ is a vital electron carrier and an essential coenzyme in cyanobacteria and other organisms. It is generated through several conserved pathways present in all living organisms, with variations among different species. There are 3 main conserved pathways: de novo pathway, the Preiss–Handler pathway, and the Salvage pathway (Yusri et al. 2025). The de novo pathway, found in bacteria, plants, and mammals, starts with aspartate or tryptophan and produces quinolinic acid (QA), a precursor to NAD^+^ (Houtkooper et al. 2010; Ishikawa and Kawai-Yamada 2019; Amjad et al. 2021). The Preiss–Handler pathway, found in bacteria, yeast, and mammals, uses nicotinic acid (NA) as a precursor, which is converted into nicotinic acid mononucleotide (NaMN) by nicotinic acid phosphoribosyltransferase (NAPRT) and eventually into NAD^+^ (Gazzaniga et al. 2009). The salvage pathway, found across all domains of life, recycles nicotinamide (NAM) or nicotinamide riboside (NR) to produce NAD^+^ via nicotinamide mononucleotide (NMN) by nicotinamide nucleotide adenylyltransferase (NMNAT) (Denu 2007). Cyanobacteria, such as *S. elongatus* PCC 7942 possess genes that indicate the presence of both de novo and salvage pathways (Gerdes et al. 2006). However, the specific contribution of each pathway under different physiological conditions (such as light versus dark and stress conditions) has not yet been studied. Additionally, the putative NAD^+^ biosynthetic enzymes in cyanobacteria are not fully characterized.

Cyanobacteria are good model organisms for studying NAD(H) metabolism. First, as unicellular prokaryotes, cyanobacteria lack the multiple organelles found in plant cells, which prevents the compartmentalization of NAD(H) functions, making their study more straightforward. In contrast, the presence of organelles such as chloroplasts and mitochondria in plants complicates NAD(H) function by distributing it across different cellular compartments (Steinbeck et al. 2020). Second, the respiratory chain in cyanobacteria is primarily located on the cytoplasmic membrane, with some components shared with the photosynthetic electron transport chain on the thylakoid membrane (Lea-Smith et al. 2016). This integration improves metabolic efficiency and flexibility, enabling most metabolic reactions to occur within a single cellular compartment. Consequently, cyanobacteria can quickly adapt to environmental fluctuations, such as transitions from darkness to light (Santos-Merino and Ducat 2023).

In this study, we found that the NAD^+^ concentrations in *S. elongatus* PCC 7942 exhibited diurnal fluctuations during the light–dark cycle, with a reduction during the dark phase. Simultaneously, a homolog of NMNAT in cyanobacteria, NMNAT-C, was found to exhibit increased levels when NAD^+^ levels decrease. We experimentally confirmed that NMNAT-C participates in the NAD^+^ salvage pathway and plays an important role in sustaining NAD^+^ homeostasis in the dark phase. Investigating the mechanisms behind NAD^+^ oscillations by studying NMNAT-C's physiological roles in the NAD salvage pathway enhances our understanding of NAD(H) metabolism in cyanobacteria. This will provide valuable guidance on manipulating NAD^+^ metabolism for synthetic biology applications in cyanobacteria, as well as insights into NAD^+^ metabolism in higher plant species.

## Results

### Rhythmic fluctuations in NAD^+^ and *NMNAT-C* expression levels in *S. elongatus*

**Figure 1:**
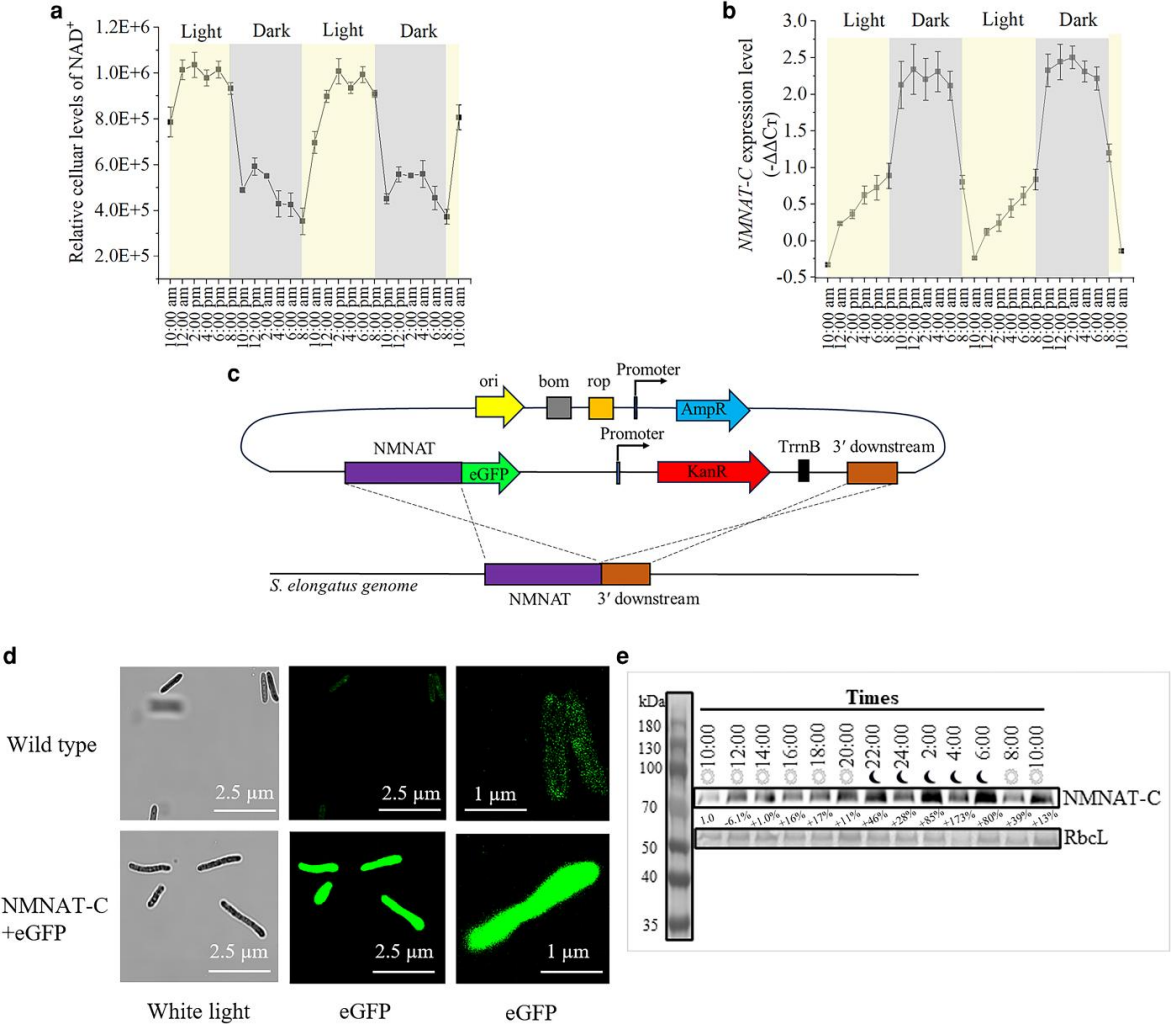
Rhythmic fluctuations in NAD^+^ and NMNAT-C expression levels in *Synechococcus elongatus.*  **a)** Rhythmic fluctuations of NAD^+^ levels in *S. elongatus* in the diurnal cycle. Data represent the mean ± SD (*n* = 3). **b)** RT-qPCR analysis of *NMNAT-C* expression levels in *S. elongatus* in the diurnal cycle. Data represent the mean ± SD (*n* = 3). **c)** Schematic representation of making an eGFP fusion through double homologous recombination. The enhanced fluorescent protein (eGFP) tag was fused to the 3′ end of *NMNAT-C*, followed by the kanamycin (kanR) resistance cassette. **d)** Confocal microscopy images of eGFP-tagged NMNAT-C in *S. elongatus* in vivo. Left: white light; right: GFP emission. **e)** Western blot analysis of NMNAT-C protein levels in *S. elongatus* every 2 h during a full dark–light cycle. Ribulose bisphosphate carboxylase large chain (RbcL) served as an internal reference protein.

We first determined NAD^+^ levels under light–dark cycles. One mL culture of *S. elongatus* was collected every 2 h during 2 light–dark cycles, and NAD^+^ was extracted and quantified by High-Performance Liquid Chromatography coupled with Tandem Mass Spectrometry (HPLC-MS) analysis. The NAD^+^ measurements revealed diurnal-like rhythmic fluctuations, with elevated levels during the light phase and reduced concentrations during the dark phase (Fig. 1a). To reveal the mechanism behind the NAD^+^ fluctuation in the diurnal cycle, the expression pattern of key genes in the NAD^+^ biosynthetic pathways was examined by RT-qPCR. Notably, the *NMNAT-C* homolog expression exhibited an inverse pattern to that of NAD^+^ changes (Fig. 1b). Spearman's rank correlation analysis revealed a significant negative association between NMNAT-C expression and NAD^+^ levels (ρ & −0.58, *P* < 0.005). We introduced an enhanced green fluorescent protein (eGFP) tag by fusing it to the 3′ end of the *NMNAT-C* in the genome through double homologous recombination using kanamycin as a selection marker during the transformation process (Fig. 1c). Confocal imaging of the GFP fusion protein confirmed successful in-frame insertion of eGFP, indicating that NMNAT-C is a cytoplasmic protein with a uniform distribution (Fig. 1d, column 3). The levels of the fusion protein were then assessed by Western blotting using anti-eGFP antibodies. The results showed that NMNAT-C protein levels also exhibited diurnal fluctuations, similar to their transcript levels, but with a delayed peak and larger fluctuations (Fig. 1e). This discrepancy suggests that NMNAT-C may be subject to post-translational regulation, whereby protein abundance is shaped by dynamic control of protein stability, degradation, and post-translational modifications.

### NMNAT-C exhibits NMN adenylyltransferase activity dependent on a conserved H/TXGH motif

**Figure 2:**
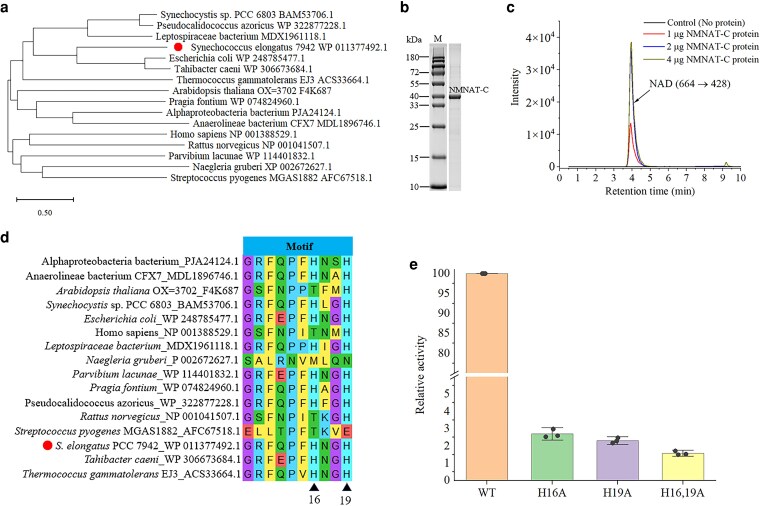
The characterization of the NMNAT-C protein in *Synechococcus elongatus* PCC 7942. **a)** Phylogenetic trees of NMNAT proteins constructed using the neighbor-joining method with MEGA X (Kumar et al. 2018). **b)** Purified recombinant NMNAT-C shown in SDS-PAGE. **c)** In vitro validation of the NMN adenylyl transferase activity of NMNAT-C. The activity was assessed by mass spectrometry through the detection of NAD^+^ production from NMN and ATP. **d)** The multiple sequence alignment of a conserved motif from NMNAT proteins of various species is displayed. Triangles denote 2 histidines (H16 and H19) selected for generating mutants. Amino acids are color-coded according to their side-chain chemistry. **e)** Comparison of the activity of wild-type and mutants of NMNAT-C with a single-point mutation (H16 or H19 to Ala) and 2 single-point mutations (both H16 and H19 to Ala). The results represent the average of 3 independent experiments, with error bars indicating ± SD. M, protein molecular weight marker (kDa).

Many NMNAT family proteins from a wide range of prokaryotic and eukaryotic species have been characterized (Magni et al. 2004). As shown in Fig. 2a, the NMNAT-C from *S. elongatus* PCC 7942 clusters closely with NMNATs from other bacteria, indicating a high degree of sequence conservation among prokaryotic NMNATs, while eukaryotic NMNATs form distinct, more divergent branches. This phylogenetic analysis indicates that NMNAT-C from *S. elongatus* PCC 7942 shares a conserved evolutionary origin with bacterial NMNATs. To determine if the cyanobacterial NMNAT-C can catalyze NAD^+^ formation by functioning as an NMN adenylyltransferase, we expressed and purified NMNAT-C as a 6xHis-tagged recombinant protein (Fig. 2b). The purified NMNAT-C was incubated with the substrates NMN and ATP, and the reaction products were analyzed by mass spectrometry. A peak with a mass-to-charge ratio (m/z) identical to that of the authentic NAD^+^ standard was detected (Fig. 2c), confirming NAD^+^ production. Furthermore, the conversion efficiency of NMN to NAD^+^ exhibited a protein concentration-dependent manner (Fig. 2c), demonstrating that NMNAT-C can catalyze the formation of NAD^+^ from NMN and ATP.

The alignment of NMNAT protein sequences from diverse organisms reveals a highly conserved motif, characterized by the presence of critical residues, including 2 conserved histidines (corresponding to positions 16 and 19 in the cyanobacterial NMNAT-C) (Venkatachalam et al. 1999). This motif is present across bacteria, cyanobacteria, archaea, amoeba, and mammals and plants (Fig. 2d), underscoring its evolutionary conservation and likely functional importance. To assess the role of these histidine residues in catalysis, site-directed mutagenesis was performed to individually substitute His16 and His19, as well as both residues simultaneously, with alanine. Enzymatic assays demonstrated that mutation of either histidine substantially reduced its NMN adenylyltransferase activity, whereas the double mutant (H16,19A) exhibited almost complete loss of activity (Fig. 2e). These results indicate that both conserved histidine residues are essential for enzymatic function, which have been reported to be likely involved in stabilizing structural motifs, metal coordination, or catalysis within the active site of the enzyme (D’Angelo et al. 2000).

### NMNAT-C is essential for NAD^+^ homeostasis in the dark phase in *S. elongatus*

**Figure 3:**
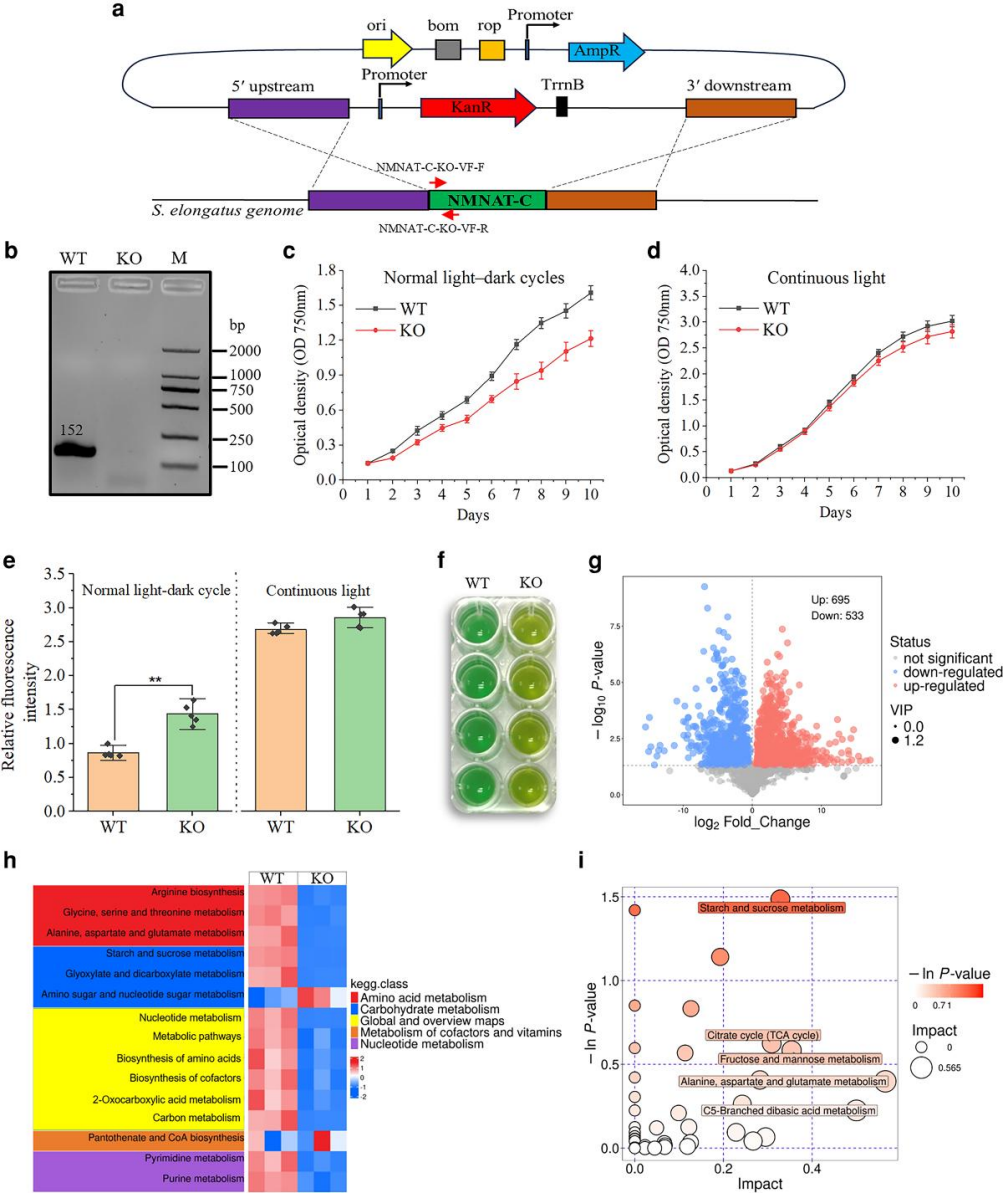
Physiological effects of NMNAT-C knockout on *S. elongatus* PCC 7942. **a)** Schematic diagram illustrating the process of making the knockout mutation through double homologous recombination. The vector construct includes an origin of replication (ori), the mobility region from pBR322 (bom), the 1,000 bp homologous arms flanking the NMNAT-C gene, and the cassette containing the kanamycin gene. **b)** DNA gel electrophoresis confirming the successful knockout of the *NMNAT-C* gene. **c, d)** Growth curve of the *NMNAT-C* knockout strain (KO) and wild-type strain (WT) over 9 days of culture under normal light–dark cycle and continuous light conditions. Data represent the mean ± SD (*n* & 4). **e)** Cellular reactive oxygen species (ROS) levels in wild-type and *NMNAT-C* knockout strains under normal light–dark cycles and continuous light conditions at the end of the 9-day growth rate testing experiment. *y*-axis values represent relative fluorescence intensities normalized per protein concentrations. ** *P* < 0.01. **f)** The *NMNAT-C* knockout strain exhibits a yellowish phenotype. **g)** Volcano plot illustrating the differentially expressed metabolites in wild-type and mutant strains. **h)** KEGG pathway-enrichment heatmap of differential metabolites between the wild-type and mutant strains. **i)** Bubble plot showing significantly enriched pathways. M, DL2000 Plus DNA Marker.

To further investigate the physiological functions of NMNAT-C in *S. elongatus*, the gene was knocked out through homologous recombination (Fig. 3a). The recombinant plasmid was introduced into *S. elongatus* cells at the logarithmic growth phase via natural transformation and selected on plates containing kanamycin resistance. Cyanobacteria have multiple copies of chromosomes per cell. Therefore, mutants were screened by gradually increasing antibiotic concentrations. The complete segregation of mutants was confirmed through PCR (Fig. 3b).

To compare the growth properties of the knockout line with WT, the same initial biomass of cyanobacteria (OD_750_ & 0.15) was grown in BG-ll medium, and their biomass was measured on different days of growth. During 9 days of cultivation under normal light–dark cycles, significant differences in biomass (OD750) were observed, indicating growth inhibition caused by *NMNAT-C* deletion (Fig. 3c). The growth inhibition was also validated by measuring colony-forming unit (CFU) using plating assays (Figure S1). We also measured the growth performance of the 2 strains under continuous light conditions (Fig. 3d). A slight, but not significant, decrease in growth rate was observed in the knockout line, suggesting that NMNAT-C contributes to maintaining NAD^+^ levels specifically during the dark phase. Reactive oxygen species (ROS) levels in the knockout and WT lines were quantified using the H_2_DCFDA fluorescence assay at the end of the 9-day growth rate testing experiment. A significant increase in ROS levels was observed in the NMNAT-C knockout strain under normal light–dark cycles, whereas no significant difference was detected under continuous light conditions (Fig. 3e). Additionally, after 2 months of continuous cultivation under normal light–dark cycles, the *NMNAT-C* knockout strain showed a yellowish phenotype (Fig. 3f).

Cell cultures of the wild-type and mutant strains were collected and subjected to nontargeted metabolomic analysis (De Vos et al. 2007; Zhou et al. 2022). Compared with the wild type, 533 metabolites were upregulated and 695 metabolites were downregulated in the *NMNAT-C* mutant (Fig. 3g). KEGG pathway-enrichment analysis of these differential metabolites revealed that the *NMNAT-C* knockout strain generally had lower levels of metabolites related to amino acid, carbohydrate, and nucleotide metabolism (Fig. 3h). Among them, starch and sucrose metabolism showed the most substantial changes. Other prominently-enriched pathways included the citrate cycle (TCA cycle), fructose and mannose metabolism, and C5-branched dibasic acid metabolism (Fig. 3i). This suggests that knocking out *NMNAT-C* disrupts these metabolic pathways, leading to significant changes in central metabolic processes compared with the wild-type strain.

**Figure 4:**
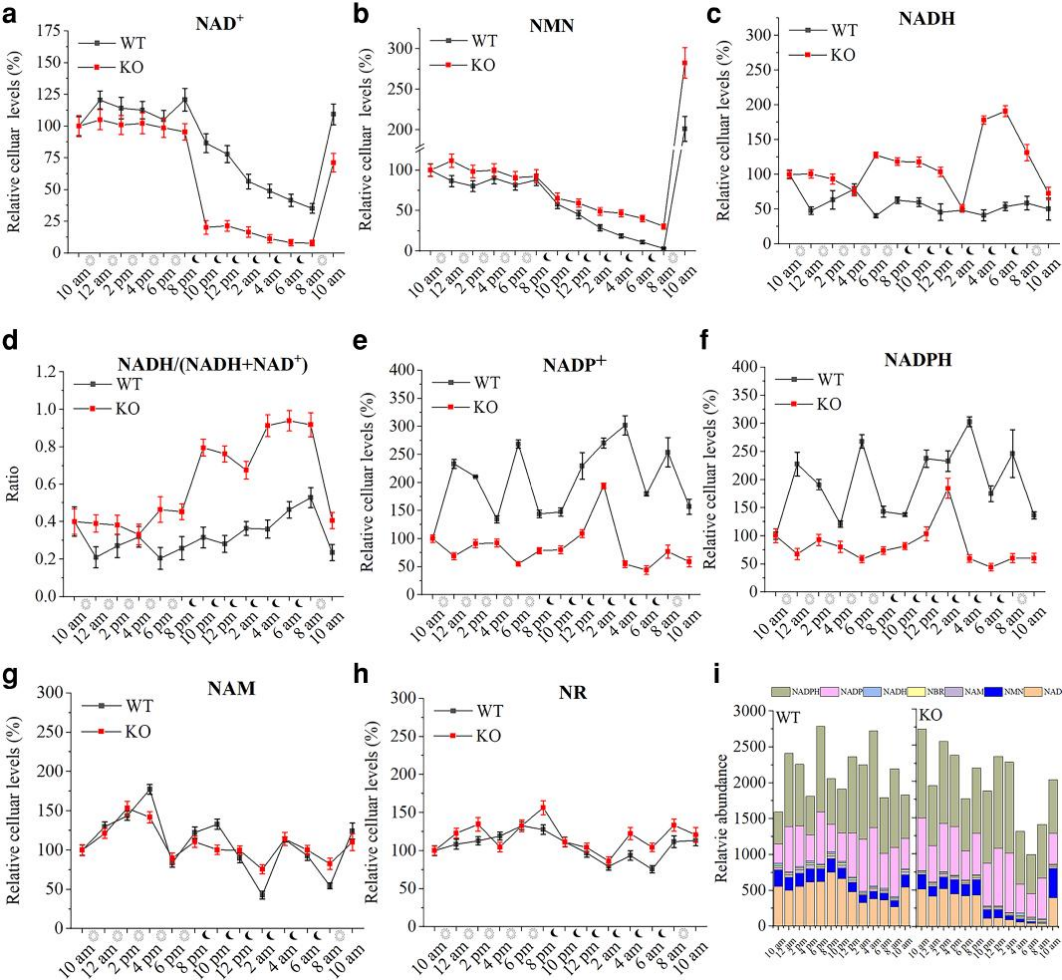
Effect of NMNAT-C knockout on the cellular NAD^+^ levels and its related metabolites during the light–dark cycle. **a** to **h)** Changes of levels of NAD^+^  **(a)**, nicotinamide mononucleotide (NMN) **(b)**, NADH **(c)**, the ratio of NADH/(NAD^+^ and NADH) **(d)**, NADP^+^  **(e)**, NADPH **(f)**, nicotinamide (NAM) **(g)**, nicotinamide riboside (NR) **(h)** NAM during a whole light and dark cycle in wild-type (WT) and *NMNAT-C* knock out (KO) strains. Data represent the mean ± SD (*n* & 3). **(i)** Total levels of NAD^+^ and these related metabolites are shown in **(a)** to **(h)**. Values are presented as mean from 3 biological replicates.

We compared the change of NAD^+^ levels at 2-h intervals throughout a complete light–dark cycle in the WT strain and the *NMNAT-C* knockout strain. In the WT strain, the NAD^+^ concentration remained at relatively high levels during the light cycle but declined rapidly upon switching to the dark cycle, continuing to decrease as the dark phase progressed. When re-exposed to light, NAD^+^ levels quickly rebounded (Fig. 4a). A similar pattern was seen in the *NMNAT-C* knockout strain, except NAD^+^ levels declined more rapidly during the dark phase and became extremely low by the end (Fig. 4a). The result shows that NMNAT-C helps maintain NAD^+^ levels during the light-to-dark transition and prevents them from dropping too sharply and becoming too low at the end of the dark phase. Meanwhile, NMN levels in the *NMNAT-C* knockout strain declined more slowly than in the WT strain during the dark phase (Fig. 4b). This aligns with the functional role of NMNAT-C, which uses NMN as a substrate to synthesize NAD^+^. Together with the results presented in Fig. 1 and the known physiological functions of NMNAT-C, these findings suggest that NMNAT-C should play a role in maintaining NAD^+^ homeostasis through the NAD^+^ salvage pathway during the dark phase.

Fluctuations in NAD^+^ levels are influenced by its synthesis, utilization, and conversion into other metabolites. To better understand these dynamics, we also analyzed the patterns of key NAD^+^-related metabolites, including NADH, NADP^+^, NADPH, nicotinamide (NAM), and nicotinamide riboside (NR) (Fig. 4c to i). In the *NMNAT-C* knockout strain, NADP(H) displayed a clear reduction. Unlike NAD^+^, these metabolites did not exhibit rhythmic fluctuations during the light–dark cycle. The trend in NAD ^+^ -related metabolites indicates that the decline in NAD^+^ levels during the dark phase is unlikely to result primarily from its conversion to NADH or NADP(H) but is more likely due to its consumption in uncharacterized biological processes in cyanobacteria.

### Overexpression of NMNAT-C disrupts NAD^+^ homeostasis and impairs cyanobacterial growth

**Figure 5:**
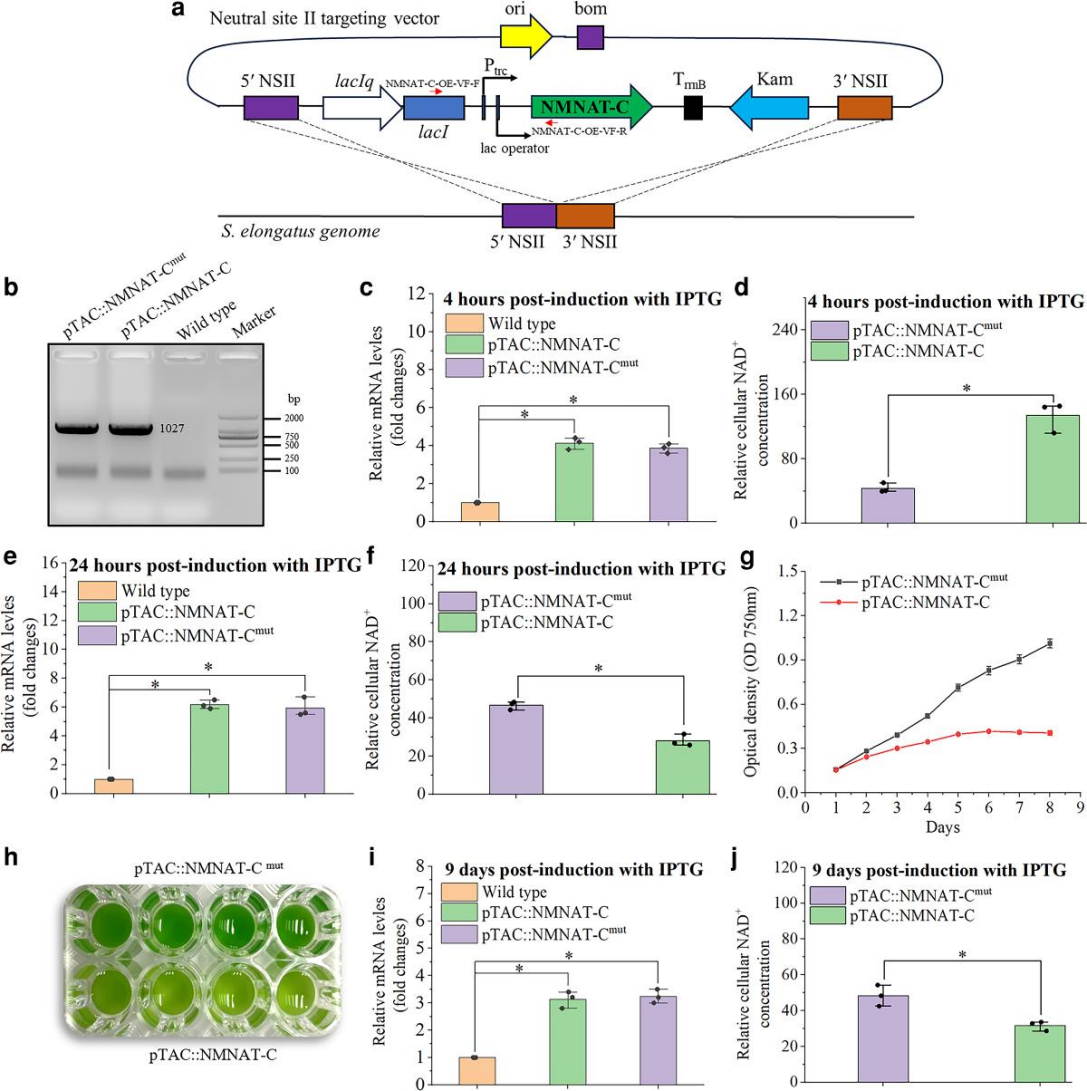
Physiological effects of NMNAT-C overexpression in *S. elongatus* PCC 7942. **a)** A schematic diagram illustrating generation of the overexpression strain through double homologous recombination and the vector constructs, including an origin of replication (ori), the mobility region from pBR322 (bom), the neutral site I (NSI), the trc promoter (Ptrc), lacIq promoter, lac repressor (lacI), lac operator, the cloned target gene *NMNAT-C*, and the cassette containing the kanamycin gene. The arrows indicate the genomic regions amplified by the primers used to validate the overexpression strains. **b)** Using DNA gel electrophoresis to confirm insertion of the overexpression construct. pTAC::NMNAT-C refers to the strain that overexpresses NMNAT-C. pTAC::NMNAT-C^mut^ refers to the strain overexpressing a mutated version of NMNAT-C used as a control. **c, e, i)** RT-qPCR was performed to evaluate NMNAT-C expression levels after IPTG induction for 4, 24 h, and 9 days, respectively. **d, f, j)** Relative cellular NAD^+^ concentrations after 4, 24 h, and 9 days of IPTG induction, respectively. **g)** Growth curves of the 2 strains over 9 days of culture. **h)** The NMNAT-C overexpression strain appeared to have a yellowish phenotype. Statistical analysis was conducted using an independent samples *t*-test. The results show the average of 3 independent experiments, with error bars indicating ± SD. *P < 0.01.

The physiological function of NMNAT-C was further investigated by overexpressing the enzyme in vivo. The *NMNAT-C* overexpression cassette was integrated into a neutral site of the *S. elongatus* genome to construct an IPTG-inducible overexpression strain (pTAC::NMNAT-C) (Fig. 5a). Meanwhile, another overexpression strain, carrying both H16A and H19A mutations, was also constructed and served as a control strain (pTAC::NMNAT-C^mut^). DNA Gel electrophoresis confirmed the successful insertion of the *NMNAT-C* overexpression constructs (Fig. 5b). Following induction with 0.1 mmol IPTG at 12:00 AM for 4 h, the mRNA levels of *NMNAT-C* in the overexpression strain and the control strain increased by about 4-fold when compared with the wild-type strain indicating success in building the IPTG-inducible constructs (Fig. 5c). Associated with the increased expression of NMNAT-C was about a 3-fold rise in cellular NAD^+^ levels in the overexpression strain (pTAC::NMNAT-C) compared with the control strain (pTAC::NMNAT-C^mut^) (Fig. 5d). However, after 24 h of induction, despite a further rise in *NMNAT-C* mRNA levels were detected (Fig. 5e), cellular NAD^+^ levels in the overexpression strain declined by about 2-fold in comparison with the control strain. Results from an 8-day growth experiment further confirmed that prolonged high *NMNAT-C* expression negatively impacts the growth of *S. elongatus* (Fig. 5g). Prolonged *NMNAT-C* overexpression caused about a 3-fold decrease in final biomass of the overexpression strain compared with the control strain after 9 days of cultivation, along with a visible color change in the *NMNAT-C* overexpression strain (Fig. 5h). The mRNA level of *NMNAT-C* in the overexpression strain was 3-fold higher than that of the control strain after 9 days of IPTG induction (Fig. 5i) but with a 35% decrease in NAD^+^ concentration (Fig. 5j).

**Figure 6:**
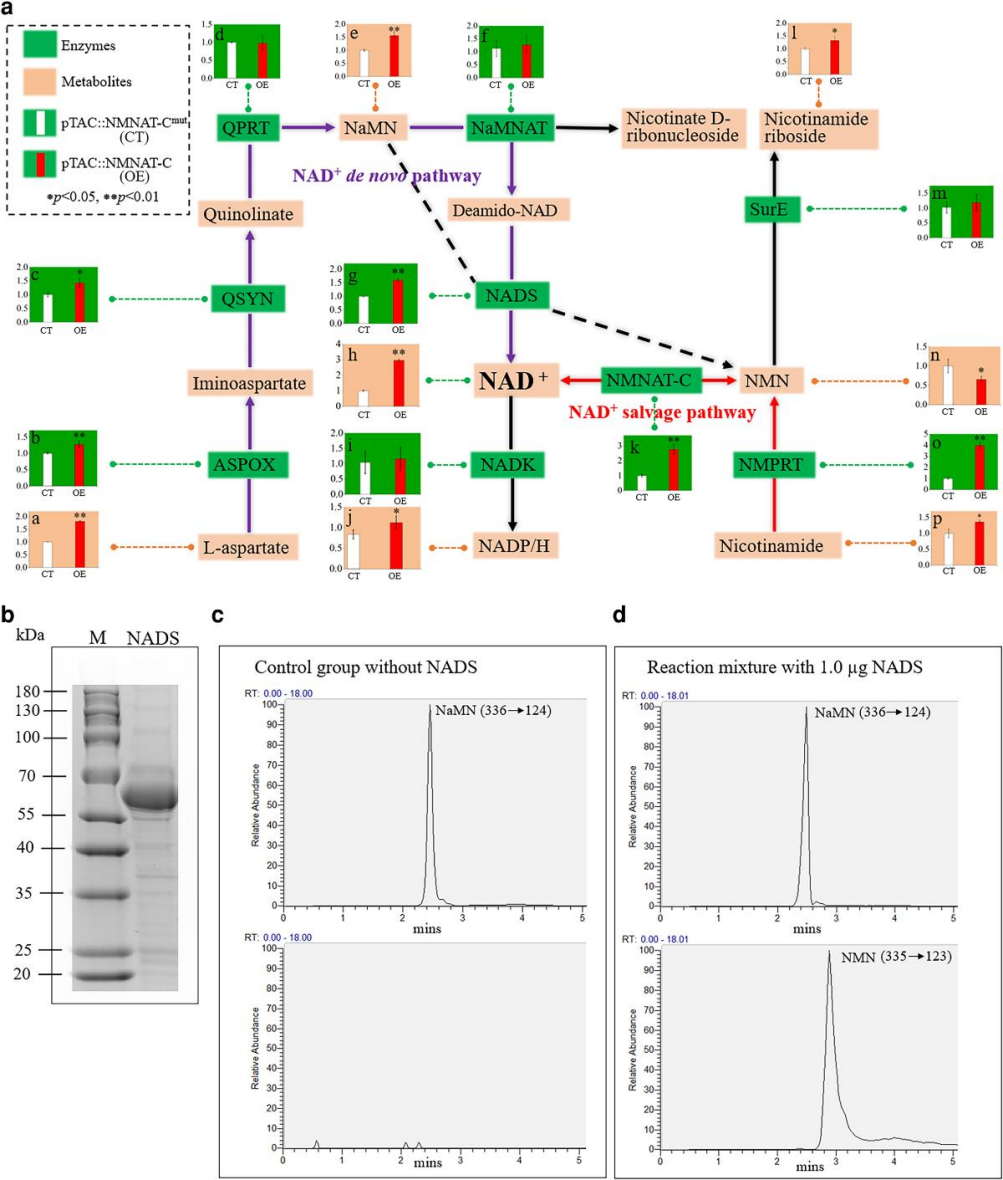
The effects of NMNAT-C overexpression on the NAD^+^ synthesis pathways in *S. elongatus* PCC 7942. **a)** NAD^+^ synthesis pathways in *S. elongatus* PCC 7942. In the NAD^+^ de novo pathway (purple line), L-aspartate is first oxidized to iminoaspartate by L-aspartate oxidase (ASPOX), which is then converted to quinolinate, nicotinate mononucleotide (NaMN), nicotinic acid adenine dinucleotide (Deamido-NAD), and NAD^+^ in sequence by a series of enzymes. In the salvage pathway, nicotinamide (NAM) is first transferred to nicotinamide mononucleotide (NMN) by nicotinamide phosphoribosyl transferase (NMPRT), which is then converted to NAD^+^ by nicotinamide nucleotide adenylyltransferase (NMNAT-C). NADK is NAD^+^ kinase; QSYN is quinolinate synthetase; QPRT is quinolinate phosphoribosyl transferase; NaMNAT is nicotinate nucleotide adenylyltransferase; NADS is NAD^+^ synthase; CinA is nicotinamide nucleotide amidase; and SurE indicates 5′/3′-nucleotidase. The column figures show RT-qPCR results (green background) and the concentrations of intermediate metabolites (orange background). The white column represents the control group (CT), while the red column represents the group (OE) involving NMNAT-C overexpression induced by 0.1 mmol IPTG for 4 h. Enzymes contributing to NAD^+^ synthesis are highlighted in green squares, and the *y*-axes indicate the relative expression levels of the enzymes. The intermediate metabolites are highlighted in orange squares, and the *y*-axes indicate the relative concentrations of the metabolites. The dark dashed line indicates a putative conversion of NMN to NAD^+^ catalyzed by NADS. **b)** Purified recombinant NADS shown in 12% gradient SDS-PAGE. Lane M, molecular weight standards; Lane S, NAD synthetase (5 μg). **c, d)** HPLC-based analysis of the NMN product catalyzed by NADS. Independent samples t-tests were utilized for 2-group comparisons. The results are the average of 3 independent experiments, with error bars showing ± SD. pTAC::NMNAT-C: IPTG-induced NMNAT-C overexpression strain, pTAC::NMNAT-C^mut^: IPTG-induced mutated NMNAT-C overexpression strain. *P < 0.05. **P < 0.01. M, protein molecular weight marker (kDa).

To clarify how NMNAT-C overexpression affected metabolic flux related to NAD^+^ metabolism, a targeted metabolomic analysis was performed after induction with 0.1 mmol IPTG at 12:00 AM for 4 h to measure key metabolites involved in NAD^+^ synthesis pathways. At the same time, RT-qPCR was conducted to assess the expression levels of key genes involved in NAD^+^ metabolism (Fig. 6a). Consistent with the finding from the in vitro enzyme activity assay that NMNAT-C is the enzyme that catalyzes the conversion of NMN and ATP to NAD^+^ (Fig. 2c), the intracellular NMN concentration significantly decreased (*P* < 0.05) in the NMNAT-C overexpressed strain when compared with the control strain (Fig. 6a to n). This reduction indicates increased NMN utilization as a substrate by NMNAT-C. Overexpression of NMNAT-C also influenced the expression level of the gene coding for another enzyme in the NAD^+^ salvage pathway, nicotinamide phosphoribosyltransferase (NMPRT) (Fig. 6a to o). NMPRT catalyzes the conversion of nicotinamide (NAM) to nicotinamide mononucleotide (NMN), which constitutes the first step of the NAD^+^ salvage pathway. Its upregulation may serve to compensate for increased NMN consumption by enhancing the conversion of NAM to NMN.

Overexpression of NMNAT-C also affected the expression of key genes in the NAD^+^ de novo pathway. The significantly increased expression of genes for L-aspartate oxidase (ASPOX) (Fig. 6a to b), quinolinate synthetase (QSYN) (Fig. 6a to c), and NAD^+^ synthase (NADS) (Fig. 6a to g) indicates that the NAD de novo pathway was upregulated. However, according to negative feedback regulation, elevated NAD^+^ levels should inhibit de novo NAD^+^ synthesis as described by the current annotated pathways (Gerdes et al. 2006; Campagna and Vignini 2023). It is possible that the upregulation of these key enzymes in the NAD^+^ de novo pathway is not primarily for NAD^+^ synthesis but mainly for NMN production to offset its depletion through the NAD^+^ salvage pathway. It has been reported that *Francisella tularensis* can directly convert NaMN to NMN via NMN synthetase ftNadE* and then the NMN is used for NAD^+^ synthesis through the NAD^+^ salvage pathway (Sorci et al. 2009). However, this pathway has not been reported in cyanobacteria. Therefore, homologs of ftNadE* in *S. elongatus* PCC 7942 were searched, and NADS was identified as a potential homologous protein (Figure S2).

The NADS is involved in the de novo synthesis of NAD^+^ by converting deamido-NAD^+^ to NAD^+^, utilizing NH_3_ as the source of nitrogen (Rizzi et al. 1996). Since the ability of NADS to convert NaMN to NMN has not been reported in cyanobacteria (Fig. 6a, dark dashed line), we purified the NADS as a 6xHis-tagged recombinant protein and conducted in vitro enzymatic assay to determine whether an alternative NMN biosynthetic pathway exists (Fig. 6b). HPLC-coupled mass spectrometry (HPLC-MS) was used to analyze the reaction products. In vitro enzymatic tests support our hypothesis that NMN could be synthesized from NaMN by NADS in *S. elongatus* (Fig. 6c and d). The finding indicates that when NMN is insufficient, the NMN biosynthetic pathway may serve as an alternative source of precursors for subsequent NAD^+^ synthesis through the NAD^+^ salvage pathway. These results indicate that the NAD^+^ biosynthesis pathway is more complex than previously recognized. Overall, these findings suggest that overexpressing *NMNAT-C* not only affects the NAD^+^ salvage pathway but also interferes with de novo NAD^+^ synthesis, suggesting that NMNAT-C should be precisely regulated to maintain NAD^+^ homeostasis in *S. elongatus*.

## Discussion

### The roles of NMNAT-C in NAD^+^ homeostasis in *S. elongatus*

Maintaining NAD(H) homeostasis is crucial for normal physiological function and is tightly regulated through various mechanisms, including biosynthesis, consumption, and recycling (Beeler et al. 2014; van Roermund et al. 2016). The salvage pathway has been known to play an essential role in maintaining NAD^+^ homeostasis in various organisms. The main salvage pathway begins with NAM transforming into NMN via NMPRT. NMN then undergoes adenylation to form NAD^+^, mediated by NMNAT (Denu 2007). The mammalian nicotinamide mononucleotide adenylyl transferase NMNAT1-3 is recognized as a key enzyme in the NAD^+^ salvage pathway, which influences NAD^+^ levels in subcellular compartments (Cambronne et al. 2016). In plants, the salvage pathway is crucial for maintaining NAD^+^ levels amid changing metabolic needs. Plants not only recycle NAM through NMNAT (or similar routes) but also combine multiple interconnected salvage branches to regulate NAD^+^ homeostasis across different subcellular compartments flexibly (Lu et al. 2025). However, enzymes of the NAD^+^ salvage pathway and their roles in NAD^+^ homeostasis in cyanobacteria remain largely unknown. In this study, the NMNAT homolog NMNAT-C was identified in *S. elongatus* 7942. We demonstrate that NMNAT-C is an NAD^+^ salvage pathway enzyme crucial for maintaining NAD^+^ homeostasis in cyanobacteria.

In addition to determining the enzymatic activity of the cyanobacterial NMNAT-C in vitro, we investigated its physiological roles. The knockout of NMNAT-C in *S. elongatus* resulted in a more rapid decrease in cellular NAD^+^ levels during the dark phase in comparison with wildtype (Fig. 4a). Conversely, induced overexpression of NMNAT-C temporarily raised NAD^+^ levels but also impaired growth and metabolic processes over time (Fig. 5). Our findings indicate that precise regulation of the *NMNAT-C* expression according to cellular metabolic states is critical for NAD^+^ homeostasis, normal metabolism, and proper growth in *S. elongatus*.

### NAD^+^ feedback regulation by NMNAT-C maintains NAD^+^ homeostasis in *S. elongatus*

Based on the dynamic patterns of NAD^+^, NMN, and NMNAT-C during the light–dark cycle, along with the biological role of NMNAT-C, we suggest that NMNAT-C in *S. elongatus* may be regulated by negative feedback by NAD^+^. During the light phase, the de novo NAD^+^ biosynthesis pathway was active, resulting in high and sufficient intracellular NAD^+^ levels. At the same time, the NAD^+^ precursor NMN accumulated via the NADS-mediated pathway (Fig. 6a, dark dashed line). Under these conditions, the expression of NMNAT-C was suppressed, leading to a decrease in the activity of the NAD^+^ salvage pathway. Conversely, during the dark phase, de novo NAD^+^ biosynthesis is insufficient for ongoing metabolic activities that consume NAD^+^, resulting in a decrease in cellular NAD^+^ levels. This decline might be sensed by cells to upregulate expression of the *NMNAT-C* gene. The increased NMNAT-C then enhances NAD^+^ production via the NAD^+^ salvage pathway, utilizing the accumulated NMN as substrate. Through this feedback mechanism, NMNAT-C plays a role in maintaining NAD^+^ homeostasis during the dark phases.

### NMNAT-C function links NAD^+^ homeostasis to stress responses in *S. elongatus*

In addition to function in redox reactions, NAD^+^ is involved in many other cellular activities such as modifications of proteins and RNAs that play important roles in cell division, stress responses, aging, and cellular signaling (Houtkooper et al. 2010; Yaku et al. 2018; Amjad et al. 2021). NAD^+^ is a substrate for protein polyADP-ribosylation, with NAM produced as a byproduct (Hottiger et al. 2010). In plants, NAD(H) homeostasis has been widely studied for its influence on growth, metabolites, nutrient assimilation, stress response, and defenses (Dutilleul et al. 2005; Sienkiewicz-Porzucek et al. 2010; Bernhardt et al. 2012; Pétriacq et al. 2012; Pétriacq et al. 2016). It has been reported that NAD^+^ status in leaves is critical for nitrate assimilation in *Nicotiana sylvestris* (Dutilleul et al. 2005). A mutation in the *Arabidopsis* quinolinate synthase gene elevates NAD^+^ levels and increases the respiration rate, accompanied by upregulated expression of oxidative stress markers, leading to early senescence phenotypes (Schippers et al. 2008). Although NAD^+^ synthase overexpression did not increase NAD^+^ levels in *Arabidopsis*, it significantly disrupted NAD^+^ redox homeostasis, resulting in ectopic ROS generation, premature wilting of the flower stalk, and reduced plant longevity (Hashida et al. 2016). However, the functions underlying NAD^+^ homeostasis in cyanobacteria remain largely unknown.

In *S. elongatus*, the *NMNAT-C* knockout strain exhibited a more rapid and pronounced decline in NAD^+^ concentration during the dark phase (Fig. 4a). This depletion of NAD^+^ compromised cyanobacterial physiological functions. After 9 days of cultivation, the *NMNAT-C* knockout strain exhibited impaired growth and a significant elevation in ROS levels (Fig. 3c and e). A clear reduction in NADP(H) was also observed in the *NMNAT-C* knockout strain, suggesting that the alteration of the NAD^+^ level might affect cellular redox states and could also affect photosynthetic metabolism (Fig. 4e and f). This demonstrates that maintaining NAD^+^ levels above a critical threshold is crucial for maintaining normal physiological functions. In addition, prolonged *NMNAT-C* overexpression caused reduced growth and a yellowish phenotype, suggesting reduced photosynthetic pigments that may affect photosynthesis. Maintaining NAD^+^ homeostasis is likely essential not only for normal growth but also for alleviating various environmental stresses, such as salinity, heat, ultraviolet radiation, and high-light intensity, which are common challenges in the industrial cultivation of microalgae in open ponds. Understanding the mechanism that maintains NAD^+^ homeostasis through the NAD^+^ salvage pathway and other pathways could provide essential knowledge for developing techniques to boost industrial production of blue-green algae.

## Materials and methods

### Strains and culture conditions

Cyanobacteria (*S. elongatus* PCC 7942) (UTEX 2434) was obtained from the UTEX Culture Collection of Algae at the University of Texas at Austin. The strain was maintained in BG-ll medium under controlled conditions at 30 °C, with agitation at 150 rpm (HiPoint 500SRC, Taiwan) and a light intensity of 50 μE photons m^−2^ s^−1^, adhering to a 12:12 light–dark cycle. *Escherichia coli* DH5a competent cells (Thermo Fisher Scientific) were employed for gene cloning and vector construction. The cells were cultivated in Luria-Bertani (LB) broth base, supplemented with specified antibiotics (100 μg/mL ampicillin, 50 μg/mL kanamycin) to select transformants and prepare the vector.

### Construction of vectors and strains

The plasmid containing a neutral site II integration platform for *S. elongatus* PCC 7942 was a gift from Susan Golden (Adeane plasmid # 40420). A modified plasmid was constructed by integrating an overexpression system under the control of Isopropyl β-D-1-thiogalactopyranoside (IPTG) into plasmid # 40420 (Fig. 5a). The NMNAT-C was cloned by PCR from *S. elongatus* PCC 7942 genomic DNA using NMNAT-EcoRI-F and NMNAT-SalI-R primers. After digestion with EcoRI and SalI, NMNAT-C was ligated into the modified plasmid. Site-directed mutagenesis was employed on the key active sites to obtain the mutant NMNAT-C overexpression vector. The overexpression strains were constructed through natural transformation (Golden et al. 1987). Briefly, 4.0 mL of exponentially growing cells were centrifuged at room temperature at 5000 × *g* for 5 min. The cell pellet was washed with a 10.0 mM NaCl solution and centrifuged again to collect the cells. The resulting pellet was resuspended in 300 µL of sterile BG-ll medium. Subsequently, the cells were incubated with 200 ng of plasmid DNA in 1.5-mL microcentrifuge tubes overnight in the dark with gentle agitation. Following incubation, the cells were exposed to light for 4 to 6 h before being plated onto selective agar plates.

The knockout of NMNAT was achieved through homologous recombination and the simultaneous introduction of a selection marker, kanamycin. DNA fragments of approximately 1,000 bp, both upstream and downstream of the NMNAT gene, were cloned into a vector, and a cassette containing the selectable marker was inserted between these flanking regions (Fig. 3a). Following PCR amplification of the recombinant plasmid fragments, multiple-fragment assembly was performed by Gibson Assembly (Gibson et al. 2010). Enhanced GFP (eGFP) was fused to the 3 “end of the NMNAT-C gene with the same method (Fig. 1e). Due to multiple copies of the chromosome in *S. elongatus* PCC 7942, PCR and sequencing verified transformants were maintained in BG-ll medium supplemented with 50 μg/mL kanamycin for several generations until complete segregation. Primers used for vector constructions and verifications are listed in the Supplementary data (Table S1).

### Expression and purification of recombinant NMNAT-C proteins

To obtain the NMNAT-C protein, BamHI and SalI sites were introduced into the NMNAT-C gene by PCR using genomic DNA as the template. The fragments were then ligated into the pET-28b vector (Novagen, Darmstadt, Germany). The recombinant plasmid was first amplified by transforming it into TOP10 Competent Cells (Invitrogen) for cloning and then transformed into *E. coli* BL21 (DE3) (Invitrogen) for protein expression. Expression was initiated by adding 1.0 mM IPTG to the medium when the optical density (OD) reached 0.6, followed by incubation for 18 h at 16 °C (Nallamsetty and Waugh 2007). The cells were harvested and resuspended in a protein buffer (50 mM Tris-HCl, pH 8.0, 100 mM NaCl) for extraction, with all steps conducted at 4 °C. The cells were disrupted by ultrasonication, then centrifuged at 13,000 × *g* for 20 min to eliminate cell debris. The resulting supernatant was applied to the His Trap column (GraviTrap™) equilibrated with the protein buffer containing 10 mM imidazole and shaken at 4 °C for 1 h. After washing with the protein buffer containing 40 mM imidazole, the proteins were eluted with the protein buffer containing 250 mM imidazole (Krynická et al. 2019). The eluted proteins were desalted using protein preservation buffer (50 mM Tris-HCl, pH 8.0, 100 mM NaCl, 4.0 mM MgCl_2_, 2.0 mM DTT) through an ultracentrifugal filter (Amicon®). The purified recombinant proteins were stored in preservation buffer with 20% (v/v) glycerol at −80 °C for subsequent analysis.

### Sampling methods

Cyanobacterial samples were collected every 2 h to study metabolite and NMNAT-C expression patterns during light–dark cycles, with cell density recorded. At each sampling point, cells were harvested by centrifugation (13,000 × *g* for 2 min), quickly quenched in liquid nitrogen, and stored at −80 °C to prevent metabolic changes. For dark-phase sampling, collections were done under light-avoidant conditions to maintain physiological integrity. For consecutive days of dark incubation, the test used 48-well plates. Wild-type and NMNAT-C mutant strains were harvested during their logarithmic growth phase via centrifugation under standard conditions. The cells were resuspended in fresh BG-ll medium, and the optical density was adjusted to OD_750_ & 0.6. A 2 mL portion of each suspension was transferred to individual wells, with 5 biological replicates per group. The plates were then wrapped in aluminum foil to block light while allowing air circulation. After 3 days of dark incubation, optical density was measured, and intracellular ROS levels were quantified.

### Detection of total ROS in *S. elongatus* PCC 7942

ROS levels in cyanobacterial cells were measured using 2′,7′-dichlorodihydrofluorescein diacetate (H_2_DCFDA) (Rajneesh et al. 2017). In brief, cells from each well of the 48-well plate were harvested by centrifugation at 5,000 × *g* for 5 min at room temperature, washed once with phosphate-buffered saline (PBS), and resuspended in 100 μL PBS containing 10 μM H2DCFDA. After incubating in the dark for 1 h, 100 μL of the supernatant was transferred to a black 96-well plate. Fluorescence intensity was measured with a microplate reader at an excitation wavelength of 485 nm and an emission wavelength of 530 nm. Protein concentrations were determined using the Bradford assay, and fluorescence values were normalized to protein content.

### Mass spectrometry

Ultra-high performance liquid chromatography (UHPLC) utilizing the Thermo Scientific™ UltiMate™ 3,000 system coupled with a Triple-Stage Quadrupole Mass Spectrometer (Thermo Scientific™ TSQ Quantiva™) was employed to quantify target metabolites in NAD synthesis pathways. Intracellular metabolites were extracted using 70% methanol containing 0.1% formic acid, 1.0 mM DTT, and 10.0 mM EDTA, while sonication facilitated cell lysis. An ultracentrifugal filter (Amicon®) removed proteins before applying the sample to HPLC-MS/MS. Mobile phases consisted of a 5.0 mM NH_4_OAc solution (A) and pure methanol (B), facilitating analyte elution at a flow rate of 0.1 mL/min. A 5 μL injection volume was chromatographically separated using a C18 column (ACQUITY UPLC BEH, 2.1 mm × 100 mm, 1.7 μm particle size) with an 18-min gradient from 5% to 15% buffer B over 2 min, and from 15% to 100% buffer B over 8 min. The column was washed for 4 min in 100% buffer B before equilibration in 5% buffer B for 4 min. A selective reaction monitoring (SRM) assay was conducted for quantitative analysis of targeted metabolites (Cao et al. 2020). The mass-to-charge ratios of precursor and fragment ions, ionization modes (positive and negative), and collision energy settings were summarized in the Supporting Information (Table S2).

Nontargeted metabolomics analysis was conducted using the Ultimate 3,000 UHPLC coupled with a Q-Exactive Focus Orbitrap mass spectrometer (Thermo Fisher Scientific, USA) in both positive and negative ionization modes. Metabolite separation was achieved with an ACQUITY UPLC HSS T3 column (2.1 mm × 100 mm, 1.8 µm, Waters Corporation, Milford, MA, USA). MS1 acquisition was performed within a scan range of 70 to 1,000 m/z, with a resolution of 70,000 and an AGC target set to 1 × 10^^6^. For MS^2^ acquisition, the specified parameters included a resolution of 17,500, an isolation window of 0.4 m/z, and collision energies set at 10, 20, and 40 C (De Vos et al. 2007). The raw data were converted to the mzXML format using ProteoWizard and processed with an in-house program developed in R, based on XCMS, for feature detection, extraction, alignment, and integration. The R package and the in-house database were used for metabolite identification (Zhou et al. 2022).

### *In vitro* enzyme activity assay

To assess the NMN adenylyltransferase activity of NMNAT-C, 100 μL reaction mixtures were prepared containing 50 mM HEPES (pH 8.2), 5.0 mM MgCl_2_, 1.0 mM ATP, 1.0 mM NMN, and varying concentrations of NMNAT-C protein (1, 2, or 4 μg). The assay for NMN synthesis activity of NADS was conducted by adding 1.0 μg of purified recombinant NADS into a 200 μL reaction system (50 mM Hepes pH 7.5, 5.0 mM MgCl_2_, 1.0 mM NaMN, 1.0 mM ATP, 2.5 mM NH_4_Cl). The mixtures were incubated at 37 °C for 1 h, then the reaction was stopped by rapid cooling and removing enzymes with an ultracentrifugal filter (Amicon®). NaMN, NMN, and NAD^+^ were analyzed using UHPLC coupled with a triple quadrupole mass spectrometer. Mobile phases consisted of 5.0 mM NH_4_OAc (A) and pure methanol (B), enabling analyte elution at a flow rate of 0.1 mL/min. A 5 μL injection volume was chromatographically separated on a C18 column (ACQUITY UPLC BEH, 2.1 mm × 100 mm, 1.7 μm particle size) with an 18-min gradient: from 5% to 15% buffer B over 2 min, then from 15% to 100% buffer B over 8 min. The column was washed with 100% buffer B for 4 min before re-equilibration in 5% buffer B for 4 min. A SRM assay was performed in ESI + mode.

### RT-qPCR and western blot

RNA extraction to assess the mRNA levels of NAD^+^ metabolism-related genes and the NMNAT-C gene by RT-qPCR was performed as described in Kim et al. (2006). The cDNA was synthesized using the PrimeScript RT Reagent Kit (Takara) according to the manufacturer's instructions. The RT-qPCR was conducted on a QIAquant qRT-PCR system (Qiagen, Hilden, Germany) with TB Green Premix Ex Taq II (Tli RNase H Plus). The *SecA* (Synpcc 7942_0289) was used as an internal reference gene (Luo et al. 2019). The RT-PCR primers are listed in the Supplementary data (Table S1).

For western blot, the crude extracts of cyanobacterial cells were obtained by breaking the cells with 0.3 mm steel beads in protein lysis buffer [50 mM Tris (pH 7.5), 150 mM NaCl, 1.0 µM DDT, 1.0 mM PMSF, 5.0 mM MgCl_2_, 1% NP-40, and 25% glycerol] containing 1% protease inhibitor (SIGMAFAST Protease Inhibitor Cocktail Tablets, EDTA-Free, Sigma-Aldrich), and the supernatant was collected by centrifugation at 13,000 × *g* for 20 min. Before the Western blot analysis, protein concentrations were determined using the BCA assay to ensure equal protein loading across samples. Proteins were fractionated using 10% sodium dodecyl sulfate polyacrylamide gel electrophoresis (SDS-PAGE) and transferred to a PVDF membrane (Immobilon-P, IPVH00010, Millipore, Burlington, MA, USA). Blots were blocked in tris-buffered saline phosphate containing 0.1% (v/v) Tween-20 and 5% (w/v) nonfat dry milk and incubated with eGFP antibody (Invitrogen) at a dilution of 1:5,000. The Western Blot Signal Enhancer (Pierce™) detected different antigens with secondary anti-rabbit antibodies (1:25,000; Sigma-Aldrich).

### Confocal fluorescence microscopy

Live-cell confocal fluorescence imaging was performed on an inverted Confocal FLIM Imaging System (Nikon C2si Plus) with a 100× oil-immersion objective (numerical aperture: 1.45). To immobilize cells for imaging, 10 µL of culture was pipetted onto an agar surface, allowed to dry, and the resulting agar section was excised and placed in contact with a coverslip. GFP and chlorophyll signals were detected using a 488 nm laser, a 525 nm filter, and a 700 nm filter, respectively. The DAPI (4′,6-diamidino-2-phenylindole) fluorescence signal was detected using a 355 nm laser and a 450 nm filter. Images were recorded by averaging each scan line 8 times. The confocal pinhole was set to give a *z*-axis resolution of about 2 µm.

### Statistical analysis

Statistical analysis was carried out using IBM SPSS 24.0. Independent samples t-tests were utilized for 2-group comparisons. Data are presented as mean ± SD, with *P* < 0.05 considered statistically significant.

### Accession numbers

Sequence data from this article can be found in the GenBank/EMBL data libraries under accession numbers. Genome accession of *S. elongatus* PCC 7942: NC_007604.1. Accession numbers of *NMNAT-C* and NADS genes: *SYNPCC7942_RS01040* and *SYNPCC7942_RS00540*. Accession numbers of NMNAT-C and NADS proteins: Q31RT2 and Q31S32.

## Supplementary Material

kiag143_Supplementary_Data

## Data Availability

The data underlying this article are available in the article and in its online supplementary material.
