# Supplementary material for: The NAD salvage pathway enzyme NMNAT-C sustains dark-phase NAD+ homeostasis in cyanobacteria
Source: Plant Physiol. 2026 Mar 16;200(3):kiag143. doi: 10.1093/plphys/kiag143 (PMC13036487; doi:10.1093/plphys/kiag143)
Supplement: kiag143_Supplementary_Data [file kiag143_supplementary_data.pdf]

Supplementary Data

The NAD Salvage Pathway Enzyme NMNAT-C Sustains Dark-Phase NAD$^+$ Homeostasis in Cyanobacteria

Feng Zhang[1#], Hailei Zhang[2,3#], Pengxi Wang[2], Yinyao Qi[2], Huankai Li[1], Lin Zhu[1], Gefei Huang[1], Yiji Xia[2,3,4,5*], Zongwei Cai[1,6*]

1 Department of Chemistry, Hong Kong Baptist University, Hong Kong SAR, China

2 Department of Biology, Hong Kong Baptist University, Hong Kong SAR, China

3 College of Biological and Environmental Sciences, Zhejiang Wanli University, Ningbo, 315100, China

4 State Key Laboratory of Agrobiotechnology, The Chinese University of Hong Kong, Hong Kong SAR, China

5 AoE Centre for Plant Vacuole Biology and Biotechnology, The Chinese University of Hong Kong, Hong Kong SAR, China

6 Eastern Institute of Technology, Ningbo 315200, China

# authors contributed equally to this work

*Corresponding authors: Yiji Xia (yxia@hkbu.edu.hk); Zongwei Cai (zwcai@eitech.edu.cn)

The author responsible for distribution of materials integral to the findings presented in this article in accordance with the policy described in the Instructions for Authors (https://academic.oup.com/plphys/pages/General-Instructions) is Zongwei Cai.

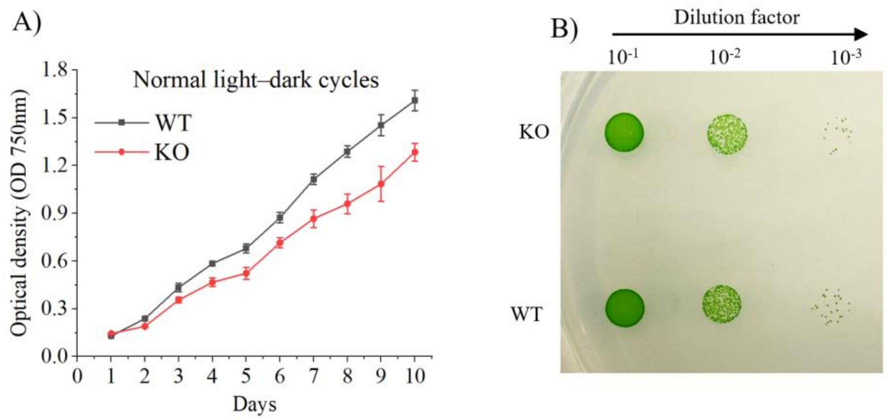

Supplementary Figure S1. Physiological effects of NMNAT-C knockout on *S. elongatus* PCC 7942. A) Growth curve of the *NMNAT-C* knockout strain (KO) and wild-type strain (WT) over 9 days of culture under normal light-dark cycle. Data represents the mean ± SD (n = 4). **B)** Cell densities of the NMNAT-C knockout (KO) and wild-type (WT) strains were assessed at the end of the 9-day cultivation experiment by colony-forming unit (CFU) counts using plating assays.

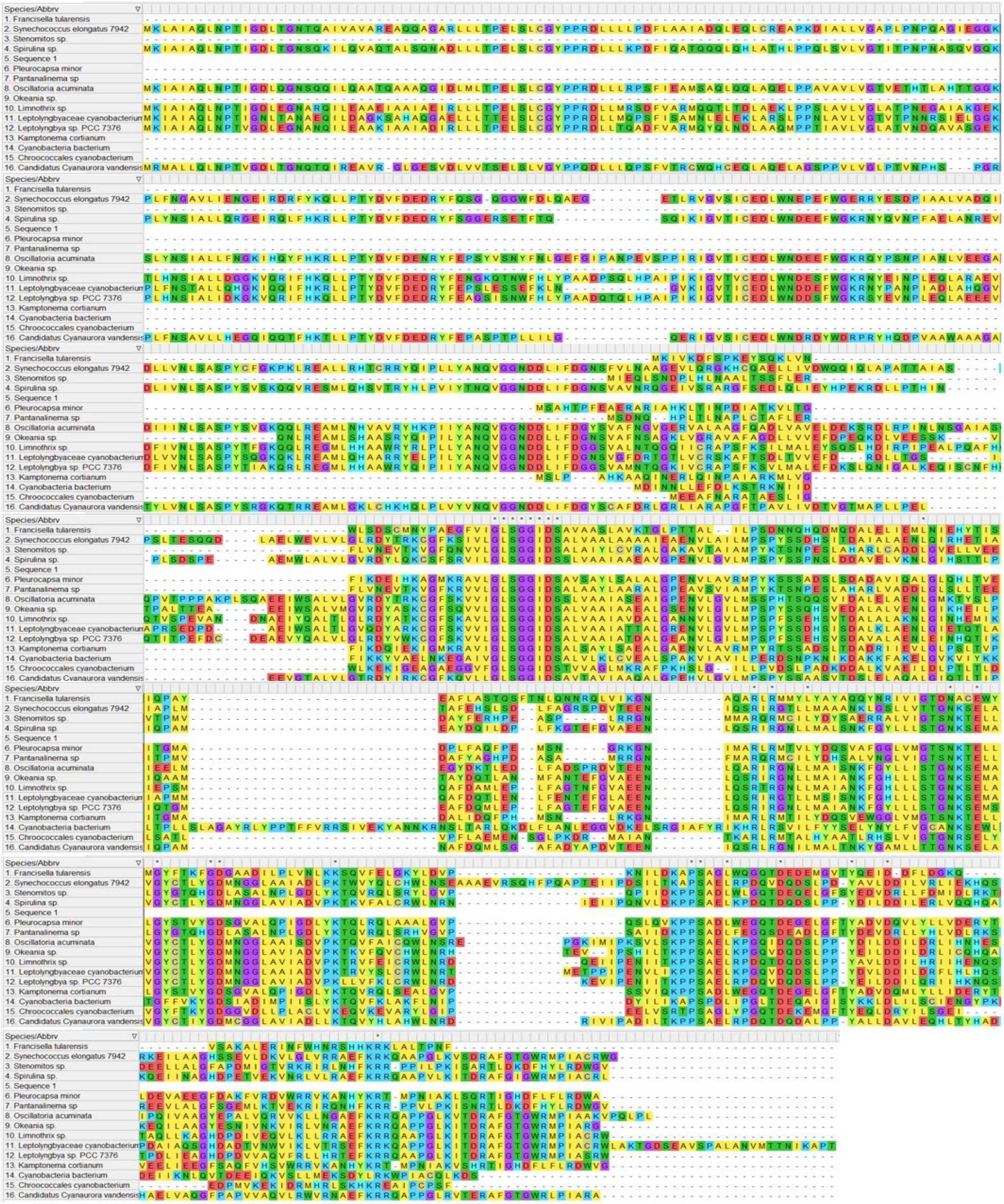

Supplementary Figure S2. Homologs of ftNadE* identified in cyanobacterial species. Multiple sequence alignment was performed using ClustalW (Kumar et al., 2018). Amino acids are color-coded according to their side-chain chemistry. Blue (KR); Red (E, D); Yellow (F, Y, W); Green (N, T, C, Q, S); Orange (P, A, V, M, L, I, G); White (any / gap).

Supplementary Table S1. List of plasmids, primers and DNA oligos used in this study

**Plasmids used in this study/ sources**	
pET-28a (+)	Novagen, https://www.merckmillipore.com/AU/en/product/pET-28a-DNA-Novagen,EMD_BIO-69864
Plasmid #40240	Addgene, https://www.addgene.org/40240/
Plasmid #40248	Addgene, https://www.addgene.org/40248/
NMNATC-KO	Modified from Plasmid #40240
NMNATC-OX	Modified from Plasmid #40248
NMNATC-eGFP	Modified from Plasmid NMNATC-KO
**Primers**	**5' > 3' sequences**
Primers for constructing modified plasmids in this study	
NVNSII-F	ACGGGTAACCGATATCGCGCGCGCGAAGG (forward)
NVNSII-R	GGGTCTAGAGGCCTGATGCCTGGCAGTTCCCTAC (reverse)
NVBB-F	CCACGTTGCCGTAGACCAGT (forward)
NVBB-R	CGAAGCGGGTCACTACTTGG (reverse)
Primers for constructing the NMNAT-C overexpression vector	
NMNAT-C-EcoRI-F	ATAGAATTCATGAAATACGACGTTGCGG (forward)
NMNAT-C-SalI-R	ATAGTCGACCTAAGTTCGACTCACAAAATGC (reverse)
Primers for NMNAT-C Site-directed mutagenesis	
NMNAT-C-M-F	CCAGCCATTCGCTAACGGGGCTCTGTCGGTCGTCCAACGTTCATTGG (forward)
NMNAT-C-M-R	CCGACAGAGCCCCGTTAGCGAATGGCTGGAAGCGTCCGATGTATACTGC (reverse)
Primers for NMNAT-C knockout	
NMNAT-C-HM-F-1	AGTTTGTGGGTCATTCCATGGCTTGCTCGACGCCGACCG (forward)
NMNAT-C-HM-R-1	GTCTAGAGGCCTGTCGACGATAATTGACCTCAGAACCCCTGAAGTG (reverse)
NMNAT-C-HM-F-2	TCGTCGACAGGCCTCTAGAC (forward)
NMNAT-C-HM-R-2	CGCTGAGGTCTGCCTCGTGA (reverse)
NMNAT-C-HM-F-3	TCACGAGGCAGACCTCAGCGTGCTGCTGAGATCAGTGTTAGGT (forward)
NMNAT-C-HM-R-3	AACCGCACCTGTGGCGCCGGCCGATTGCAAAATGCGATCGC (reverse)
NMNAT-C-KO-BB-F	CCGGCGCCACAGGTGCGGTT (forward)
NMNAT-C-KO-BB-R	CCATGGAATGACCCACAAACTGCTCAAAC (reverse)
Primers for constructing the NAD synthase expression vector based on pET-28a (+)	
Gibson-EcoRI-NADS-F	TGGGTCGCGGATCCGAATTCATGAAACTGGCGATCGCGCAAC (forward)
Gibson-SalI-NADS-F	GCCGCAAGCTTGTCGACTTAGCCCCAACGACAAGC (reverse)
Primers for constructing a vector for inserting eGFP into the 3' end of the NMNAT-C	
5' NMNAT-C-F	GAGGTCAATTAATGAAATACGACGTTGC (forward)
3' NMNAT-C-R	GCTCACCAT AGTTCGACTCACAAAATGCTGAATGAT (reverse)
5' eGFP-F	GTCGAACTATGGTGAGCAAGGGCGAGG (forward)
5' eGFP-R	GCGTTTCTAACTGCAGGTCCTGAAGTTAACTAGT (reverse)
5'BB-F	GTCGTATTTCATTAATTGACCTCAGAACCCCTGAAGTG (forward)
3'BB-F	CCTGCAGTTAGAAACGCAAAAAGGCCATCCG (reverse)

Primers for RT-qPCR of genes engaged in NAD synthesis pathways	
L-aspartate-F	CGGCAGTGACAGCTCCAGATGA (forward)
L-aspartate-R	GTTCAAGCAGCGAAGCCACAC (reverse)
Quinolinate synthetase -F	GTGTTCCTCGCTGCTGATCG (forward)
Quinolinate synthetase -R	CACCAATGTAGTCCGCCACATC (reverse)
QPRT-F	CGACCCGATTCTGTCTGACTGG (forward)
QPRT-R	CGATCGGTAAGCCTGCGATG (reverse)
NaMNAT-F	ATGCATTTAGCCCTGTTTGGAACC (forward)
NaMNAT-R	GCCGCATGTTGCTTAAAAGGGTTG (reverse)
NAD^+ kinase-F	CTGCGCCAAGTGATCATCGC (forward)
NAD^+ kinase-R	CGAAGCCAGGAAAACTGGAAAGG (reverse)
NMPRT-F	CCGACTCATACAAAGTCAGCCAC (forward)
NMPRT-R	GTGATTGGCTGGAAGAAATAACGC (reverse)
SurE-F	ATGCGACTTCTGATCAGCAACG (forward)
SurE-R	GGATGGGTTTATGCAACGTCAGGC (reverse)
NMNATC-F	CGACGTTGCGGTCTACATCG (forward)
NMNATC-R	CCTCGGAACTCCAAGGATTACG (reverse)
SecA-F	ACGACGGTCAGATTGCCGAGAT (forward)
SecA-R	GCGACATTCCCTGCTGGATTAG (reverse)
CinA-F	GCCGGTGGGATTGGTCTACATC (forward)
CinA-R	GGTCAAATTCAGCAGCGATCGC (reverse)
Primers for verifying transformants	
NMNAT-C-KO-VF-F	CGACGTTGCGGTCTACATCG (forward)
NMNAT-C-KO-VF-R	CCTCGGAACTCCAAGGATTACG (reverse)
NMNAT-C-OE-VF-F	ATAGCGGAACGGGAAGGCG (forward)
NMNAT-C-OE-VF-R	TCAGTCAGCCAAAGATTGTCAC (reverse)

Supplementary Table S2. A summary of metabolite parameters for mass spectrometry analysis, including the mass-to-charge ratios of precursor and fragment ions, ionization modes (positive and negative), and collision energy settings.

Compouds	Precursor (m/z)	Daugther ions (m/z)	ionization modes	Collision energy
Aspartic acid	134	74	Positive	20ev
		88		
Quinolinate	166	122	Negative	25ev
		78		
Iminoasparate	132	72	Positive	20ev
		68		
		86		
Nicotinate mononucleotide (NaMN)	336	124	Positive	25ev
		97		
Nicotinamide adenine dinucleotide (NAD+)	664	428	Positive	40ev
		542		
		348		
Deamido-NAD (NAAD)	665	428	Positive	40ev
		348		
NADH	666	428	Positive	20ev
		232		
NADP+	742	620	Negative	50ev
		408		
		273		
Nicotinamide mononucletide (NMN)	335	123	Positive	20ev
		97		
Nicotinamide (NAM)	123	80	Positive	30ev
		96		
Nicorinamide-beta riboside	255	123	Positive	35ev
		106		
NADPH	744	408	Negative	50ev
		397		
		426		
ATP	508	348	Negative	30ev
		410		
